# Children’s Perceptions about Environmental Sustainability, Food, and Nutrition in Chile: A Qualitative Study

**DOI:** 10.3390/ijerph18189679

**Published:** 2021-09-14

**Authors:** Gabriela Fretes, Amapola Sepúlveda, Camila Corvalán, Sean B. Cash

**Affiliations:** 1 Friedman School of Nutrition Science and Policy, Tufts University, 150 Harrison Avenue, Boston, MA 02111, USA; gabriela.fretes@tufts.edu; 2 Facultad de Ciencias Sociales, Universidad de Chile, Santiago 6850331, Chile; amapola.sepulveda@ug.uchile.cl; 3 Institute of Nutrition and Food Technology (INTA), University of Chile, Santiago 7830489, Chile; ccorvalan@inta.uchile.cl

**Keywords:** schoolchildren, sustainability, food, eco-labeling, warning labels, focus groups

## Abstract

Food is inextricably linked to human health and environmental sustainability; however, very little is known about children’s perceptions of the concept of sustainability in the context of food choices. We aimed to explore the perceptions of Chilean schoolchildren about environmental sustainability, food, and nutrition. Eight online focus groups were conducted with boys and girls aged 8–9 (*n* = 30). Questions related to environmental sustainability, pocket money, and food characteristics such as price, front-of-package (FOP) warning label, and eco-labels were included. Data analysis was conducted using ATLAS.ti through a hybrid content analysis approach. Five central themes were identified: (1) children’s favorite snacks, (2) knowledge of sustainability, (3) sustainability and eco-labels use, (4) healthfulness of food products, and (5) pocket money and food prices. Most children were not aware of the meaning of “environmental sustainability”, but the concept was understood when it was explained in plain language. Participants showed awareness about the environmental impact of their eating behavior, had a positive perception of eco-labels, and identified food with fewer warning labels as “better” options. Results indicate that children understand the concept of sustainability in food if it is communicated clearly, and that eco-labels may be an effective tool in that effort.

## 1. Introduction

Diets have been identified as one of the main contributors to rising levels of child obesity and chronic disease [1]. Snacking on energy-dense nutrient poor (EDNP) foods is considered to have a great influence in children’s overweight [2,3]. In Chile, snacking contributes over a quarter of low-middle income Chilean children’s daily energy intake, including grain-based desserts, salty snacks, sweets and desserts, dairy, and cereal-based foods [4]. Although children’s diets are largely determined by their caretakers, children’s crucial role as consumers in their own rights, as well as influencers in their own households and as future adult decision makers, needs to be recognized and incorporated into our understanding of child nutrition [5].

There is growing interest in how we can make diets healthier and more sustainable [6,7,8]. According to the Food and Agriculture Organization (FAO), “sustainable diets are those diets with low environmental impacts which contribute to food and nutrition security and to healthy life for present and future generations. Sustainable diets are protective and respectful of biodiversity and ecosystems, culturally acceptable, accessible, economically fair and affordable; nutritionally adequate, safe and healthy; while optimizing natural and human resources” [9]. Part of achieving healthy and sustainable diets relies on consumer behavior, making children an important target group in part because eating behaviors developed during childhood are more likely to persist in adulthood [2,10]. Many factors influence children’s food choices: nutritional knowledge, marketing of food products, peer behavior, possession of discretionary income, and the power to influence parents’ purchases, among others [5,11,12,13]. Since children’s food choices and eating behaviors are closely linked with their preferences, and autonomy in food choices increases as children age [14], learning about environmental sustainability and its relationship to food during childhood could help shape healthier and more sustainable food choices. 

An increased awareness of climate and sustainability is driving changes in people’s food choices, modes of transportation, and consumption in general [15,16,17]. Although consumer responses to different environmental sustainability food attributes—such as “organic”, “environmentally friendly”, and others—have shown that adult consumers are becoming more ecologically conscious [18,19], this topic is understudied in children, whose knowledge of environmental sustainability and food is lacking. Most of the literature has investigated eco-friendly behaviors in adolescents and young adults [20,21,22] and one study demonstrated that information about the impact of food products on health and the environment may be difficult to process for younger Dutch children [23]. Piaget’s traditional theory of cognitive development argues that children younger than 7 years of age cannot process abstract information [24]. While some studies have looked at brand representation ability and brand symbolism to understand how children navigate and behave in the commercial marketplace using theory of mind and executive functioning [25,26], there is not much empirical evidence focused on addressing links between food sustainability and the aforementioned theories. In 2016, Chile implemented the first mandatory national front-of-package (FOP) warning label system on packaged foods high in sugars, sodium, saturated fats, and calories [27]. While it has been reported that Chilean children have become agents of change in their households by demanding healthier snacks [28] one year after the implementation of the Chilean Law of Food Labeling and Advertising [27], to date, there have been no investigations regarding what younger Chilean children understand about the concept of environmental sustainability when it is analyzed alongside other food attributes, such as the warning nutrition labels. 

With this study, we aim to qualitatively explore perceptions about environmental sustainability through focus group discussions with children. Additionally, we explored perceptions about other food attributes such as FOP warning labels, price, and product type. 

## 2. Materials and Methods

We used the Consolidated Criteria for Reporting Qualitative Research (COREQ) to guide this study’s reporting [29]. The protocol for this study was approved by both the Ethics Committee of the Institute of Nutrition and Food Technology (INTA) at the University of Chile and the Tufts Social, Behavioral, and Educational Research (SBER) Institutional Review Board.

### 2.1. Study Design

We conducted activity-based online focus group discussions (FGD) [30]. The FGD included three types of activities (i.e., drawing, writing, and picture selection) to keep children’s attention, and to create a fun and comfortable environment that enhanced discussion about the topics of interest [30,31]. For instance, we asked participants to draw their favorite snack and to write down the prices they would be willing to pay for food products shown in slides. The discussions included four parts: (1) a visualization activity, (2) pocket money discussion, (3) food attribute elicitation, and (4) environmental sustainability discussion. Data collected through the discussions will inform the design of a future online survey and choice experiment focused on the influence of sustainability and warning labels on children’s food choice.

### 2.2. Participants and Recruitment

For this study, we recruited a purposive sub-sample of children participating in the ongoing cohort study “Food Environment Chilean Cohort (FECHIC)”. The FECHIC study started in 2016 and children’s recruitment was carried out in public schools in the Santiago Metropolitan Region by the Institute of Nutrition and Food Technology (INTA) of the University of Chile in partnership with the National School Assistance and Scholarship Board (JUNAEB). Children in FECHIC are from low-to-middle-income families from the southeast area of Santiago, Chile. Additional information about the cohort can be found elsewhere [4,32]. For this study, the only inclusion criteria to participate was to be a FECHIC participant. Children’s parents were invited to participate in the study through phone calls and emails. During the phone calls, parents were informed about the specific objective of the study, potential benefits, potential risks, and the voluntary nature of the study. If parents agreed to participate, an email including a consent form and an assent form for the child was sent. We recruited the first 30 children who returned their signed parental consent and child assent forms by email. The recruiter reviewed the forms and contacted the parents through email to schedule the online FGD. The final sample of participants was 30 children from the FECHIC study.

### 2.3. Procedures

The discussions were facilitated in Spanish via Zoom in July 2020 by the first author and lasted on average 60 minutes each. Each session targeted 3–5 children. Initial rounds focused on children’s perceptions about food and nutrition and later rounds were used for food attribute elicitation and environmental sustainability discussions. Different children participated in each focus group session. The second author served as a notetaker during each discussion. As discussions were conducted online, the participants were at their respective homes, and in most cases, parents or adults were nearby.

### 2.4. Discussion Guide

All FGD were conducted using a script including semi-structured questions (Appendix A). Portions of this script were based on the one used previously by Hartman et al. 2017 [33], but the script was adapted to the Chilean context and expanded to include a greater understanding of sustainability. 

**Figure 1:**
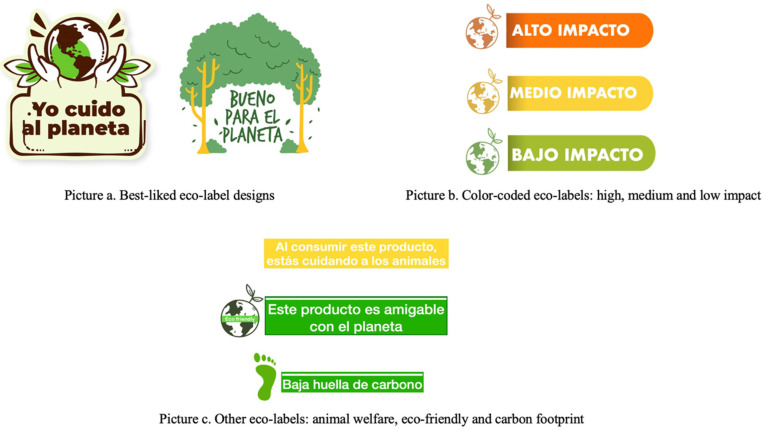
Sustainability labels designed by the investigators.

**Figure 2:**
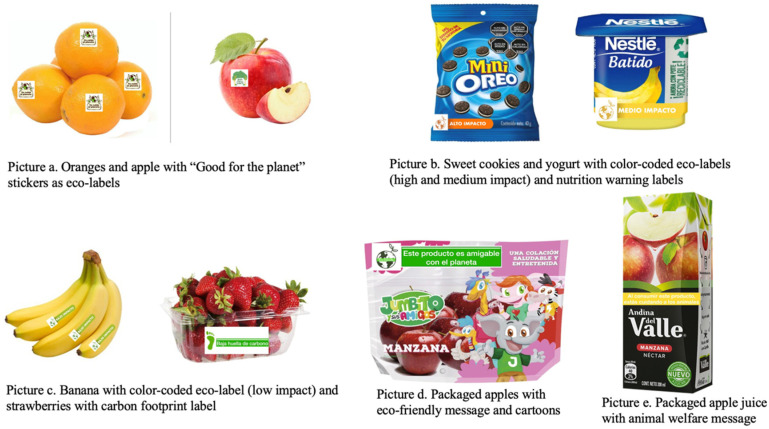
Food products with sustainability labels designed by the investigators.

At the beginning of each session, we performed a warm-up activity where participants discussed their favorite snacks and the reasons why they like them. This activity served to build rapport between researchers and children. After setting up the ground rules for the online discussion and reassuring participants that there were no right or wrong answers [34], we proceeded with the interview questions. In the first part, we conducted a visualization activity where children had to picture themselves having some money and wanting to purchase a snack such as hot dogs or empanadas, fruits or vegetables, or packaged products such as yogurt, candy, cookies, or chips during a school break. These foods were chosen for this activity because previous research reported that Chilean children usually buy them with their pocket money [35]. We asked them to draw what they chose on a paper sheet and show it on the screen. Participants had to explain what they chose, why they chose that food, and the most important feature they take into consideration when selecting a snack. In the second part of the conversations, children were asked about pocket money: whether they received money, if they used it to buy snacks, if they had to ask an adult before buying something, and where they bought snacks. The third part consisted of elicitation of attributes of interest when purchasing snacks: we showed them pictures of commonly consumed snacks with different attributes (product type, taste, brand, package, etc.), such as strawberry and banana yogurts from different brands (Soprole, Calán, Nestlé), sweet cookies (Morocha, Oreo, Quaker), fruit juices (orange, peach), fruits (apples, oranges), a salty wheat-cracker snack (Ramitas), a flavored milk (banana Yogu-yogu), and apple chips. Children were asked to choose what product they would buy, and to explain why they chose it. In this part, we also conducted an open-ended willingness-to-pay exercise where children were showed different foods and were asked to write down how much money they were willing to pay for each product. Children had to show their paper sheets with the prices on the screen. The fourth and last activity addressed environmental sustainability perceptions: we asked children if they knew the meanings of phrases such as sustainability, carbon footprint, emissions, and pollution, and whether they knew about actions to help of the planet such as reducing meat consumption and avoiding excessive packaging. We also used proxy language such as “take care of the planet” or “help the planet” to identify sustainability concepts. In the first four of our eight focus groups, we showed children eco-labels designed by the investigators and asked children if they liked the labels and what they meant to them (Figure 1). The labels included concepts such as “carbon footprint”, “good for the planet”, and “high, medium, and low impact”. In the second four focus groups, we added the best-liked eco-labels from the previous rounds to photographs of the packaging of different snacks to put environmental sustainability in the food context (Figure 2). We asked children if they liked the eco-labels, what they meant to them, and if they would be willing to buy some of the products with an eco-label. All snack images were modified from images of actual products available in Chilean grocery outlets.

### 2.5. Analyses

We used descriptive statistics to summarize participants’ socio-demographic information. Each focus group was audio- and video-recorded via Zoom (version 5.1.2) and then transcribed verbatim in Spanish. Data analysis for qualitative information was conducted in Spanish using ATLAS.ti Scientific Software (Development GmbH, Berlin, Germany, version 7.1.5 in Windows), through a hybrid content analysis approach, which combined deductive and inductive coding [36]. Two team members independently reviewed and coded the field notes. Some excerpts were translated into English by a bilingual team member. Deductive coding was made according to the topics previously created for the discussion guide: favorite snack, pocket money, the different food attributes considered when children selected a snack (e.g., preference, taste, price, brand, package characteristics), and knowledge and perceptions about environmental sustainability. Inductive coding was applied to new themes emerging from children’s responses related to environmental sustainability. Participant’s names were coded to ensure anonymity; for example, N3(5) corresponds to child 3 from focus group 5. 

## 3. Results

Eight online focus group discussions were conducted via Zoom with children (*n* = 30) aged 8 to 9 years, including both girls and boys from the Santiago Metropolitan Region, Chile. Of these participants, 56.6% (*n* = 17) were female. The mean age was 8.3 ± 0.4 years. Within this sample, three children had internet connection problems, and three decided to keep their camera off. 

The analyses identified five central themes: (1) children’s favorite snacks, (2) knowledge of sustainability concepts, (3) sustainability and eco-labels use, (4) healthfulness of food products and other food attributes, and (5) pocket money and food prices.

### 3.1. Theme 1. Children’s Favorite Snacks 

Children’s favorite snacks included sugary beverages, followed by fruits and vegetables such as apples, bananas, and oranges, and then grain-based desserts, sandwiches, and salty snacks. Most children based their choices on taste; some mentioned specific flavors (“I like strawberry yogurt”, “because it has chocolate”) or a product’s “refreshing” quality. Some children mentioned healthy eating as the main driver of choice.

*“[I would buy] juice and cookies. Juice is very refreshing. I would choose cookies with chocolate chips because they are nice.” (N2(4))*

*“[Shows a drawing of a salad] Because you have to eat healthy, fruits and vegetables, to be stronger and do sports. When I buy (snacks) I look for carrots, lettuce, and cucumber.” (N2(1))*

### 3.2. Theme 2. Knowledge of Sustainability Concepts

When we asked children about the terms “sustainability”, “carbon footprint”, and “emissions”, none of them could define the concepts. Several children mentioned hearing those words from their parents, other adults, or the news. However, when we framed the concept of sustainability as “taking care of/helping the planet”, most children could explain what this entailed. The word “pollution” was also broadly recognized and associated with throwing garbage in the streets and the sea. Children could also differentiate between different types of trash and some mentioned composting. Several identified planetary pollution as a result of other people’s actions, emphasizing that adults are responsible for planet pollution.

*“[Taking care of the planet means] not throwing garbage in the street, not throwing it in the sea, or the bottles in the garbage can because it also pollutes.” (N3(5))*

*“[Pollution] is leaving papers on the floor. Throwing fruit skin is not because it degrades and turns into soil.” (N4(8))*

*“[…] People sometimes don’t put packages in garbage bins, like this cookie package, or throw them anywhere. They don’t know how not to pollute.” (N1(6))*

### 3.3. Theme 3. Sustainability and Eco-Labels

The concept of “taking care of/helping the planet” was key to identifying sustainable behaviors and perceptions about environmental sustainability. Recycling was mentioned by most children as the main action they would implement to take care of the planet and noted different activities such as separating garbage between recyclable and non-recyclable, reducing food packaging, and making eco-bricks “to build houses”. 

*“I would take care of the planet by reutilizing plastic things and throwing things in the recycling bin.” (N2(5))*

*“The more packages, the more we pollute the Earth and the more we pollute it people get sick. Then doctors cannot help if the air is bad.” (N1(5))*

*(Showing an object) “[This is an] eco-brick. You have to find a bottle; put papers you have for recycling inside, and there it is. […] I have four, and you have to make a lot to build a house for example.” (N3(5))*

When asked if eating less meat was an action to take care of the planet, most of them responded positively and indicated animal extinction and animal welfare as the main reasons why eating less meat could help the planet. Extinction was associated with the need for “having enough” animals for future consumption. Children reported seeing in the news how animals suffer as a consequence of pollution. Additionally, when children were exposed to eco-labels with statements such as “When you consume this product, you are taking care of animals” (Figure 2e), children expressed the need to recycle packages so animals are not hurt. However, they did not associate meat consumption with sustainable production practices.

*“I think we could [help the planet if we eat less meat], because people would not kill more animals and we help animals avoid extinction.” (N4(7))*

*Asked if eating less meat helps the planet: “I think yes because we stop killing animals that suffer.” (N4(8))*

Overall, children liked the eco-labels presented on different food products. Among different types of eco-labels, a simple label in the form of a sticker was better understood. Specifically, children associated a sticker on fruits with better production practices and sustainable food consumption.

*“The sticker means that these oranges were taken care of and not damaged.” (N4(7), about Figure 2a)*

*“I like the sticker [on the oranges] because it says you’re taking care of the planet [when you eat them].” (N2(7), about Figure 2a)*

Many children had trouble interpreting the eco-label with colors indicating low, medium, and high impact when presented on its own. However, when we put the label on food packages, children seemed to have a better understanding of what the label represents by indicating that it was assessing the impact of the food product on the planet, as well as if the package was recyclable. When children compared products with a “high impact” vs. a “medium impact” label, they could differentiate which product was better for the planet and would choose the one with less impact, even if the product with the “high impact” label was their favorite snack.

*“I don’t really know because Oreos are my favorite cookies but the yogurt… I would vote for [the banana yogurt], because it’s ‘half polluted,’ because if I choose A it will be like all the world is polluted.” (N2(7), about Figure 2b)*

While overall children demonstrated understanding and positive perceptions about the eco-labels, when eco-labels were presented with food products that included a FOP warning label, there was confusion in some cases.

*“The mini-Oreos have “High impact” because they are not healthy, and the banana yogurt has fewer calories…” (N2(6), about Figure 2b)*

*“I say [food with the high impact label] is high in sugar, high in saturated fats, so it means it’s bad for you. And the yogurt is to drink and to recycle.” (N3(5), about Figure 2b)*

### 3.4. Theme 4. Healthfulness of Food Products and Other Food Attributes

Children showed positive attitudes regarding FOP warning labels and mentioned they look for FOP labels when they choose a snack to evaluate the healthfulness of the product. The importance of fruit and vegetables consumption and healthy eating was mentioned several times and highlights that children are well aware of the potential benefits of eating fruits and vegetables.

*“I always look if it’s healthy or unhealthy. [The warning label] shows me a sticker that [says] ‘unhealthy’ or ‘healthy.’ It tells me ‘it has too much sugar,’ ‘it has many bad things.’” (N1(8))*

Other food attributes such as package color, presence of cartoons, and gifts were less important for some children, but most of them expressed they are attracted to these when they select a snack.

*“I like the packages. My favorite is the Yogu-Yogu one because it is my favorite milk. I like it because of the cartoon that has the eyes and mouth open. I also like it because of the sticker.” (N2(7))*

### 3.5. Theme 5. Pocket Money and Food Prices

The majority of children reported receiving pocket money from their parents or close family and said they use the money to buy snacks at different venues: school kiosks, corner stores near home, or supermarkets. Children considered price as an important attribute when they choose a snack and recognized they usually make a decision taking into account budget constraints.

*“I look [at the price] to see if I have enough money to buy it.” (N1(6))*

*“I look [at the price] too, and if I don’t have [the money], I buy something else.” (N3(5))*

When we asked how much money they were willing to pay for some of the food products, prices reported by children generally matched the actual market price of the product. However, children who did not usually receive pocket money reported unusual values (too low or too high), showing lower familiarity with prices. Higher prices were given to products that children said they liked or were their favorites.

## 4. Discussion

The goal of this study was to explore children’s perceptions about environmental sustainability in the context of food choices in a sub-sample of children from the FECHIC study in Santiago, Chile. We further explored perceptions about other food attributes such as FOP warning labels, price, and product type. Overall, we found that while Chilean children clearly understand what a FOP warning label means for their health, they are less knowledgeable about the impact of the food they eat on the environment. In addition, there is some confusion when both FOP warning labels and eco-labels are presented at the same time on a food product.

### 4.1. Taste Is the Main Driver of Children’s Favorite Snacks

As previously published literature suggests [30,37,38], we find that taste was the main driver of children’s favorite snacks. Children’s snack preference for sugary beverages including sodas, fruit juices, and yogurts was high, which aligns with results from one study that found sugary beverages were among the top five snack consumption categories in this same cohort of children [4]. Although the concept of healthy eating was highlighted multiple times as another driver of snack choice, it is clear that the preference for healthy foods such as fruits and vegetables could have been a result of social desirability bias, given the context in which the research was being conducted (online, at home, with adults nearby), the fact that children are participants of a longitudinal cohort with a nutritional focus, and the previously reported low consumption of fruits and vegetables in this group of children [4,32].

### 4.2. Communicating Sustainability Concepts

Most children did not understand the concepts of “sustainability”, “carbon footprint”, or “emissions” and had never encountered these concepts before. One potential explanation for this finding is that younger children still cannot process complex information about abstract constructs such as the environment in contrast to their older or adolescent counterparts [23,39,40]. The average age of children in our study was 8.3 years. Our findings align with results from a Dutch study of children who were on average 8 years old and did not understand the impact of food products on their health and the environment when pop-up messages were presented to them in a virtual reality supermarket [23]. More empirical research is needed to understand the links between Piagetian stages of development, theory of mind and executive functioning, and food sustainability in children. However, when we portrayed the concept of sustainability as “taking care of/helping the planet”, children in our study established a relationship between environmental sustainability and the food they eat by associating green behaviors such as recycling packages or eating less meat to actions that help shape a more sustainable planet. Additionally, children’s worry about animal suffering shows some level of awareness about animal welfare issues, previously observed in studies of adolescents [22,41,42]. However, other topic areas such as sustainable food production or food workers’ conditions were not mentioned. Recognizing that these areas are relevant for the sustainability concept, future studies should explore how to integrate social aspects into sustainability messaging. Our findings highlight the importance of using age-appropriate terminology when communicating with children about environmental sustainability. Furthermore, it is of interest to engage young children in environmental learning as early as possible as a way to cultivate “a potentially life-long disposition of care for the environment” [43]. There are resources available to incorporate education for sustainability in the school curriculum [44,45,46]. Nevertheless, the resources may need to be adapted and validated according to the context.

### 4.3. Using Simple Eco-Labels to Inform about Environmental Sustainability of Foods

Eco-labels have been proposed as a way to communicate the potential impact of food on the environment [47]. In our study, a simple eco-label in the form of a sticker including elements typically associated with the environment (such as the planet, hands, or leaves) and a short message was well understood by children. Interestingly, when children first gave their opinions about the eco-labels alone, the most complex color-coded label was not well understood. These results are similar to what was previously found with FOP nutrition traffic light labels in the Latin American context [48]. However, when we added the label to a food product and asked children to choose between two products, most children could differentiate which product was “better for the planet”. Though the label design could have had an influence on the understanding, this result highlights the importance of presenting labels within the context where they are going to be applied and the importance of keeping messages simple and clear. To date, studies have focused on investigating perceptions about different types of eco-labels in adults and shown that people are starting to take environmental sustainability into account when they chose foods [47]. Our findings show that children have some level of awareness about the impact of their behaviors on the planet, but further research is needed to understand how this translates into actual purchasing behavior.

### 4.4. Children Use FOP Warning Labels to Assess Healthfulness of Food Products

Our study confirms that Chilean children understand the meaning of the FOP warning labels and identify foods with fewer labels as better options as compared to those with more labels. It has been documented that children living in Chile show positive attitudes about the intended messaging of the warning labels (e.g., children ask for snacks with fewer labels to take to school) and are demanding healthier snacks in their households [28]. This suggests that exposure to the warning labels at home, at school, and through different media have influenced children’s perceptions about the impact of the food they eat on their health. Future studies should evaluate how these perceptions and awareness influence food choice and consumption. Nonetheless, when the color-coded eco-label and the FOP warning label were presented together in a food product, children had a mixed understanding of the meaning of each label. This finding agrees with previously published literature suggesting that consumers may feel “overloaded” with information affecting their decision-making process and, consequently, their choices [49]; this may be even more true for children. In addition, a study conducted with Chilean adults found that nutrient claims can negatively affect consumers’ perception of product healthiness even in the presence of a FOP warning label [50]. This is important to consider when a nutrition label and an environmental sustainability label can be found in the front of a food package. Part of the confusion may come from the fact that food companies are starting to include their own eco-claims highlighting packaging properties (e.g., “recyclable package”) and not considering the sustainability of the food itself. There were other attributes, such as packaging and price, that also were mentioned as important drivers of snack choice. Future research should focus on evaluating the interactive effects of different food attributes such as price, product type, FOP warning labels, and eco-labels in an experimental framework in order to identify trade-offs and the value children give to each of the attributes mentioned. Future studies should also examine the potential effect of interventions that encourage the adoption of healthy and more sustainable food behaviors in youth and identify specific actions through which children can take part in discussions about food systems transformation.

To our knowledge, this is the first study that assessed Chilean children’s perceptions about environmental sustainability in the context of food choice. A key strength of our study was the use of innovative activity-based online focus group discussion with children, which helped us obtain in-depth information regarding the knowledge children have about the food they eat, their health, and the environment by creating a fun and comfortable environment for discussion. Furthermore, this study was conducted in Chile, an extremely appropriate setting in which to explore how FOP warning labels and eco-labels presented in packaged foods might interact, given that the current national FOP warning labeling policy has been implemented for four years. While the country has pioneered efforts on food label warning systems, if environmental sustainability messaging is to also be adopted, it must occur in a coherent and integrated way. Our study also has limitations. Focus groups were conducted with low-to-middle-income 8–9-year-old children from southeastern Santiago and our findings may not be generalizable to all Chilean children. In addition, we did not have full control over the participants given the online environment in which the focus groups were conducted. In some cases, parents were nearby, and their presence could have influenced children’s responses. Although we have observed some sort of social desirability bias in children’s responses related to health, the main researcher reminded children that “It is not like being in school because there are no right or wrong answers”. Despite the limitations, this study contributes to the literature about environmental sustainability in the context of food choices by analyzing children’s perceptions and by setting a baseline for future work in the realm of food sustainability and children’s food behavior.

## 5. Conclusions

Our study found that the children in our sample have a good understanding of the Chilean FOP warning label messaging but a low degree of awareness about the impact of their eating behavior on the planet. Children demonstrated a positive perception of eco-labels, although language communicating ideas of environmental sustainability is complex and needs further consideration. Empirical research and theories from the marketing and communication literature can be used to evaluate predictors and determinants of children’s recognition of environmental sustainability when it is associated with food products. Future work should explore how FOP labels (nutrition and eco-labels) interact with other product characteristics and with the level of food literacy in shaping children’s food choices, and to better understand how these influences form as part of the consume socialization of children. While this is the first study of which we are aware that addressed the topic of eco-labels, it may be that children’s understanding of these labels may indeed function similar to their understanding of brands as symbols in the marketplace, and there has been a lively literature connecting this to stages of child development [24,26]. Results from this study can be useful in the design of labeling policies that integrate nutrition and sustainability aspects of diets, specifically for providing coherent consumer-facing messaging.

## Appendix A

Researchers Script Focus Group Discussion	
Moderator:	Note taker:
Date:	
Start time:	End time:
Number of children:	

1. **Welcome**

*Hi, everyone! My name is (name), and (name) and I welcome you to our Zoom discussion today. Would you tell us your names as well? Let’s do it this way, tell us your name and what is your favorite snack.*

[Brief round of introduction]

2. **Purpose of Discussion and Rules**

*We are here today to talk about snacks, about what kind of snack products you like and whether you buy those sometimes with your pocket money. In our discussion today, we want to understand what you think about different kinds of snacks and why you buy one snack and not the other? We will be asking some questions and it would be great if you answer our questions as best and open as you can so that we can learn from your answers. This is a discussion between all of us, not just between all of you and us. Feel free to also speak to each other as well.*

*Before we begin, let’s go over a few basic rules. We will not be using your names attached to anything that is said in this meeting. We might share the information said today in a publication but again, we will not be using your real names connected to anything you say.*

*It’s also important that you don’t share what other children say in this meeting today with anyone outside the group. Just as we are making sure what you say is private and not to be shared, we need to ask that you all agree to do the same. This includes telling other people who was in the group today and what they said. Remember, your participation is entirely voluntary, so if at any time you do not want to participate in the focus group, you can leave.*

*It is not like being in school because there are no right or wrong answers today. We just want to hear what’s on your mind and what you have to say. Please, raise your hand to speak. So, let’s be as supportive of each other as we can even if we might disagree on something. Okay? The most important thing is that we have a good time today.*

*Our basic rules today are:*

(1) *Please remember to respect each other by not sharing what is said in the focus group with people outside of the study.*
(2) *Speak one at a time and raise your hand to speak.*
(3) *Treat each other with respect by listening and helping others feel comfortable. Remember there are no right or wrong answers.*

*Can we agree to those three rules today?* [Wait for agreement. If questions are raised, researcher explains why those rules are important.]

*Does anyone have any questions? Feel free to ask any questions during the discussion. Any questions during the focus group that you all have, we will list on this sheet (researcher show on screen) and answer them after the focus group time.*

*Great, let’s begin!*

*Note to Moderator and Note Taker: Make sure each participant has one sheet of paper and a pencil.*

3. **Interview Questions (please check off questions as you ask them)**

***Snack activity*** *: How many of you have done visualization before? Can someone tell me what it is? We are going to do a brief visualization to get started. I’ll explain it and then we’ll try it out. The way it works is that you will close your eyes — but you don’t have to — and listen to my voice and let your mind follow what I say. Okay?*

*Close your eyes if you would like, take a deep breath and relax. Imagine the bell for recess is about to ring. You have some money in your pocket and want to get a snack from the school store or the guy that sells food at the school entrance. What is your favorite snack? You could get hot meals like hot dogs and empanadas, or fruit or vegetables or packaged foods like yogurt, candy, cookies, or chips. So, what would you choose?* [Wait for a moment.] *Now, open your eyes. There is a piece of paper in front of you, please draw or write down the snack that you would choose.*

*Tell us what you have chosen.* [Each child explains what it chose, and interviewers will ask, e.g., if this is something they buy often.] *Talk about the reasons you have chosen this snack. What is most important to you when you choose to buy a snack? What things do you look for or care about?* [Wait for open responses without giving any prompts, then prompt for any of the following:

- Price
- Taste
- Attractive packaging
- Favorite brand
- Convenient, can be eaten right away
- Special (don’t get it at home)
- Nutritional value (warning labels)
- Good for the planet]

4. **Discussion of pocket money and using it for snacks:**

- *Tell us some of the reasons why you think is good to have your own money.*
- *Do you get pocket money? How often do you get pocket money?* [See what the children say: regularly, weekly, monthly, how much, from whom do you get that money?]
- *If you want to buy something from your pocket money, do you decide alone what you like to buy, or do you ask one of your parents or another adult?*
- *Do you use pocket money to buy food snacks?*
- *Talk about the place where you usually buy snacks. Why do you like to go there?*

5. **Discussion of snacks:**

*We brought you some pictures of snacks.* [Share screen with PowerPoint slide that show different foods; ask the following questions and give time for the children to react.]

- *Do you know those products? What do you think about those? Tell us if they mean something special for you and why that is.*
- *Did you ever eat any of those? Talk about the places where can you find those.*
- *Do you like to eat any of those products? If not, why not? Which one do you like most? So, one of the products is a strawberry yogurt. Do you like strawberry yogurts? Are there other flavors such as vanilla you prefer or is strawberry high on your list? What about apples? Do you like sliced apples as snack? I have here a package of cookies. Are there other cookies you prefer? Do you drink fruit juices as snack? Which one is your favorite? Why?*
- *Now let’s do a poll! See, we have some pictures here* [Share screen with PowerPoint slide that show different foods] *and ask you to indicate how much you like the different products that I will be showing. You can select from the options in the screen which one is better for you.* [Children can check the respective box in the poll and indicate whether they like the product a lot, like it, find it ok, do not like it, or do not like it at all.] [Discussion about the indicated preferences.]
- *Would you like to buy any of those products as a snack with your pocket money? If so, which one would you like to buy most?*
- *Now, if you do see the real products, would you still like your choice? If not, why not. Who of you likes the real version of the product (s)he chose more than the picture? Who is disappointed?*
- *Now assume that we would be willing to sell those products. Write on your paper what you would be willing to pay for each of those products. If you would not want to pay anything because you do not want to have one or all of those products just write a ‘0‘. Would you show us your papers on the screen?* [Discussion of prices.]
- *What do you think about the package of the products? Do you like it? What do you like? Could you recycle it?* [Wait for responses; see whether children like the package and what they like.] *Would you prefer for the cookie package another package?* [Wait for reaction; then instructor shows the children a colorful package for comparison.]

6. **Discussion of environmental sustainability:**

Now we are going to talk about what do you think about the concept of environmental sustainability. Do you know what “sustainability” means? If yes, please explain. [If not, prompt: *Have you ever heard about things people do to take care of our planet?]*

- *What does “carbon footprint” mean to you?* [If the child had no answer or said, *“I do not know”,* then ask further questions, use other proxy words.] *Have you ever heard people talking about the carbon footprint? What might they be referring to?*
- *What does it mean to help the planet?* [If the child had no answer or said, *“I do not know”*, then ask the child to think more personally.] *What do you think you could do to help the planet?*
- *Let’s think about ways we can help the planet while we choose foods. Do you think you can help the planet if you eat less meat? Do you think that by eating less packaged foods you can help the planet? Why or why not?*
- *Now, let’s do another poll! See we have some pictures here.* [Share screen with PowerPoint slide that show different foods and ask children to indicate which of the information presented they understand the best.] *Imagine that you find this information in a food package.* [Children can check the respective box in the poll and indicate whether they understand the information a lot, understand it for the most part, understand it somewhat, do not really understand it, or do not understand it at all. Examples of information to display are eco-labels, written information about carbon footprint in a box, written information, and pictures in a box, etc.](Discussion about the indicated preferences).

*We are finished with talking.*

*Thanks for coming here today to discuss with us. Did you have fun? Do you have any questions?*

*Goodbye.*
